# Simultaneous Transmission of Photonic Services over One Fiber with an ITU 100 GHz Grid

**DOI:** 10.3390/s19071601

**Published:** 2019-04-02

**Authors:** Tomas Horvath, Petr Munster, Josef Vojtech, Vladimir Smotlacha

**Affiliations:** 1 Department of Telecommunication, Brno University of Technology, Technicka 12, 616 00 Brno, Czech Republic; munster@feec.vutbr.cz; 2 Department of Optical Networks, CESNET a.l.e., Zikova 4, 160 00 Prague, Czech Republic; vojtech@cesnet.cz (J.V.); vs@cesnet.cz (V.S.)

**Keywords:** accurate time, data transmission, optical fibers, Φ-OTDR, photonic services, pulse duration, simultaneous transmission, wavelength grid

## Abstract

The increasing interest in distributed sensors and the decreasing price of optical components have led to leveraging the use of existing fiber in deployments over optical networks and more application possibilities (from seismic activity measurement to perimeter protection and tunnel fire detection). Because of the possibility of data interference in single fibers, dark fibers are used. On the one hand, optical networks are able to transfer popular services, such as streaming and data transmission, and on the other hand, special advanced services such as an accurate time, a stable frequency, and high-power optical sensor signals can be provided. In our work, we address the simultaneous transmission of an accurate time, 100 G data, and a high-power optical sensor based on Phase-sensitive optical time domain reflectometer (Φ-OTDR). The measurement setup consists of the optical fiber G.652 (7 km), G.653 (7 km), and G.655 (10 km) and a combination of G.652D + G.653 (14 km). Moreover, we also provide results for their combination. The services were transferred in single fiber with an ITU 100 GHz channel spacing grid. We performed a set of measurements with an evaluation of the BER value for data transmission affected by a high-power sensor system and accurate time values. The results confirmed our assumptions that 100 GHz spacing is not large enough, especially with the increasing power level of the sensor system. The main aim of the article is to determine whether data are disturbed with normal 100 GHz channel spacing.

## 1. Introduction

At present, the literature suggests that data transmission will keep following the current trend, i.e., the continuous increase in the amount of data being transferred. Based on the report by Cisco [1], it is clear that in 2017, ≈122 EB of data were transmitted in a month, whereas in 2018, this figure reached ≈156 EB. By 2022, this amount of data is expected to increase up to 396 EB. One of the reasons for the increasing data transfer could be a wider deployment of high-speed interfaces on backbone networks and access networks. Customers of the access network at the edge of the millennium shared ≈155 Mbps. Fortunately, today’s standards allow for sharing a transmission capacity of 10 to 100 Gbps [2,3,4,5,6,7]. To support higher transfer rates, wavelength division multiplex (WDM) technology is used.

WDM technology has been known for over 40 years [8,9,10]. Its primary purpose was to replace space division multiplex (SDM) technology. SDM used multiple fibers to separate services on a single channel. However, the spatial separation of individual services was very uneconomical. Due to developments in the field of radiation sources, more advanced techniques could be used to connect multiple services into a single fiber by using coarse wavelength division multiplex (CWDM) or dense wavelength division multiplex (DWDM) technologies [11,12]. Both of these methods are widely used today to transfer/separate multiple services in a single optical fiber. For the purpose of multiple services being present in one fiber, multiple so-called channels are used. Each of the channels uses a different wavelength. For CWDM, the wavelength spacing is 20 nm, with the first carrier at 1270 nm and the last at 1610 nm. Overall, it is possible to create 8–16 channels with a different data formats. To use all 18 channels, it is best to use the International Telecommunication Union (ITU) G.652D fiber, as this fiber are not susceptible to the wavelength range of 1360–1450 nm [13]. DWDM technology is considerably more complicated to implement because it requires use of precise laser sources of radiation that must maintain stable wavelengths to avoid the next channel being disturbed. The channel spacing is only 0.8 nm or less. The transmission rate of the channel may be 10 Gbps, and the total number of available channels ranges from 40 to 80. A great advantage is the reach of approximately 100 km without the need to amplify the transmitted signals.

Currently, data are considered to be the base unit being transmitted. The increasing amount of transferred data is also a result of services such as streaming, Internet protocol television (IPTV), and video on demand (VoD) in high definition (HD) or ultrahigh definition (UHD) that are becoming increasingly popular. Unlike commercial telco operators, the National Research and Education Network (NREN) intends to enable transmission of advanced photonic and special services. These services can include accurate time transmission [14,15,16], stable frequency [17,18,19], or real-time network service. Real-time network service is not specifically related to performance or speed but rather to strict time constraints. As an example, one can think of the transfer of surgery over the network to doctors on the other side of the globe, etc. Generally, photonic services can be defined as an end-to-end connection between two or more endpoints. These services are defined by their photonic path and allocated bandwidth. The photonic path is the path that defines where the light travels from the source to the target(s). The recovery of a photonic path is a focus of many publications today, e.g., [20,21,22]. The allocated bandwidth represents a portion of the dedicated spectrum for the photonic service over the entire photonic path.

The optical path requirements used for the transmission of time and/or frequency depend primarily on the choice of the utilized method. The two most accurate methods for time and frequency transfer do not utilize standard data packets in the network:

1. Technology based on amplitude modulation by RF signal—For this purpose, various methods have been developed. The characteristic feature of these methods is the radio-frequency (RF) modulation signal for encoding transmitted time labels. These methods assume a symmetrical transfer path in both directions, i.e., bidirectional transmission using a single fiber, and possibly suitable methods for compensating delay variations due to external influences, especially temperature. In terms of the required bandwidth, a single DWDM channel for each direction is sufficient. Standard transceivers or fiber lasers are used as the source of the optical signal.
2. Transmission of unmodulated optical signal—The subject of transmission is the unmodulated carrier signal from a stabilized laser, which is controlled by the source of a stable optical signal (e.g., an optical clock). Therefore, such a method is used only for the frequency transmission and is not utilized for the time transmission. The standard uncompensated transmission path provides stability on the order of 1 $\times \;10^{-14}$ to 1 $\times \;10^{-15}$. To increase the stability to the order of 1 $\times \;e^{-19}$, stabilizing the optical fiber noise is required, i.e., to eliminate low-frequency interference caused mainly by vibration and shaking of the optical fiber. This method has low optical channel width requirements, such that a 50 GHz DWDM channel is sufficient.

Last but not least, the use of optical fibers as a sensor [23,24,25] has also achieved ever-increasing popularity due to falling prices of components and optical fibers. The basic monitoring of perimeters is accomplished by stretching the optical fiber along a given location and by tracking changes in the local refractive index on the fiber when pressure or another force is exerted on it. Early detection of anomalies, perimeter intrusion, and localization are crucial for critical infrastructure security. With imminent sensory systems, it is necessary to ensure mutual coexistence between sensory applications and current data networks [26,27]. There are several different ways to measure acoustic vibrations near the fiber. The easiest way is analyzing polarization states in the fiber, which provides basic information that something is happening but without any other details, such as the location or the type of the event (temperature or strain changes, acoustic vibrations, etc.). Another option is to use interferometers. Their considerable disadvantage, however, is their high sensitivity; therefore, high noise levels caused by air conditioning, active elements, temperature, etc. are problematic. Moreover, in a basic configuration, two fibers must be used, and localization is not possible. The last option for evaluating changes near the fiber is to use backscattering. Reflectometry systems’ advantages are good sensitivity, the possibility of direct localization and classification, and the use of only one fiber. On the other hand, the development of reflectometry systems is complicated, and systems require sophisticated signal processing; because of the low level of the backscattered signal, it is necessary to generate high-power input pulses. Currently, reflectometric systems are the most widely used, but for measurement, dedicated optical fiber is always used, which is inefficient and uneconomical.

The rest of this paper is structured as follows. Section 2 provides an overview of the related works. Section 3 provides an overview of our measurement setup, such as the home-developed accurate time transmission system and the Φ-OTDR sensor system. Section 4 presents our measurements results. Finally, Section 5 concludes the paper.

## 2. Related Works

Accurate time, timing, or stable frequency are traditionally important in the fields of metrology, navigation, fundamental physics, and legal time keeping. Currently, they are also important for a significantly broader range of fields, e.g., sensing, Earth sciences, geodesy, astronomy, and seismology. Advances in atomic, optical, and quantum science have led to a significant improvement in the accuracy of atomic clocks. Research in the field of atomic clocks has helped to push the boundaries of basic and applied research. The concept of atomic optical time is presented in [28]. Moreover, the authors of [29] presented research focused on increasing the accuracy of the atomic clock up to 6.4 × 10${}^{-18}$. This resulted in the improvement of the International System of Units (SI) [30], the search for time variation of fundamental constants [31], the clock-based geodesy [32], and other accuracy tests of the fundamental laws of nature [29]. Traditional radio frequency methods of precise time, transmission and dissemination use satellite methods. The more accurate two-way satellite time and frequency transfer (TWSTFT) method [33] achieves subnanosecond stability; however, it requires a pair of dedicated channels on a geostationary satellite, and thus, it tends to be very expensive. The so-called common view method is considerably affordable, using one or more global navigation satellite systems such as GPS, Galileo, GLONASS, etc. [34]. However optical methods are able to deliver significant improvements in terms of stability, mainly regarding the utilization of an optical carrier with a frequency of hundreds of THz (typically 200–600 THz). They can be used for satellite communication [35] or deployed on the ground; however, the performance of the ground methods is compromised by the limited reach and stability due to weather induced attenuation or turbulence [36]. Methods using optical fibers benefit from a significantly shorter path than those of satellite-based methods and afford the possibility of active transmission stabilization. Optical time transfer methods deliver accuracy on the order of picoseconds (or even better), which is significantly better than that of standard network methods, i.e., according to the IEEE Std. 1588. Another advantage over satellite transfer is the elimination of possible jamming and spoofing attacks, antennas, and the necessity of having a clear sky view, which might be challenging to achieve in underground facilities or very large buildings. Recently, there has been increased number of theoretical studies, laboratory trials, and even field implementations of time and frequency transfer, e.g., [15,37,38,39,40,41,42,43,44]. Many works [45,46,47,48,49] have addressed simultaneous transmissions of accurate time and stable frequency. By transferring the exact time and a stable frequency, simultaneous transmission of two photonic services is realized. Next, [50] has presented a simultaneous transmission, where the timestamp transmission is implemented by phase modulation with a spread spectrum pseudorandom modulation at 20 Mchip/s. A significant portion of works report overcoming large distances using fiber shared with data transmissions [15,37,40,41,49]. At present, there are only a limited number of publications addressing the parallel involvement of further photonic services, e.g., optical fiber sensing. The works reported in [51,52,53] describe the idea of the involvement and realization of parallel optical fiber sensing; however, the discussion is limited to different types of optical fiber and the sensing system pulse duration only.

The main contribution of this work is the presentation of a practical measurement of accurate time transmission, a sensing system, and the concept of data flow in a single optical fiber or a combination of different types of optical fibers with different pulse widths for the sensor system. Importantly, the system for the transmission of accurate time was built by our team; it is not a commercial solution. Similarly, we also built the sensory system based on the phase optical time domain reflectometer (Φ-OTDR) principle. Another benefit of this work is the practical implementation of measurements based on the WDM grid. Generally, 100 GHz is considered to be a safe between-services interval (spacing). Our measurement, however, proves that such spacing is sufficient only for certain sensory systems (limited by the power of the system). By evaluating the bit error rate (BER), the degree of interaction between the photonic services and the sensor system is demonstrated. The BER value is evaluated using Coriant Groove^TM^ G30 (manufactured by Infinera Corporation, Sunnyvale, CA, USA). This device is the only commercial product in our experimental setup.

## 3. Measurement Setup

### 3.1. Φ-OTDR

Φ-OTDR is a technique that enables the detection and localization of acoustic vibrations acting on fiber. Compared to other techniques such as Brillouin OTDR, it is not necessary to generate so many high-energy pulses, and the detection is not so complicated. On the other hand, it is not possible to distinguish local temperature changes and acoustic vibrations (there is a possibility, but it is based on a very precise classification of the event). Since we have designed and developed our own (Φ-OTDR), we have also developed interferometric distributed systems based on Mach-Zehnder or Michelson interferometers, and we can compare their benefits. A sizable advantage of interferometers is their usage of low power/energy signals and hence a lower probability of interference with other signals. Even though we would consider the use of a special setup allowing the use of only one fiber and event localization, paradoxically, the greatest disadvantage of interferometers is their high sensitivity. In the case of a real network (which is considered for use), all optical fibers are terminated in rack rooms containing active devices with fans and other sources of noise that can degrade the detected signal. Based on the abovementioned information, Φ-OTDR seems to be a suitable solution for fiber infrastructure protection.

Our own developed system is based on an ultranarrow linewidth laser source from NKT Photonics that has a wavelength of 1550.92 nm and is optimized for use in fibers with active data transmission. Compared to other commercial/research solutions, we amplify the continuous wave (CW) signal using an erbium-doped fiber amplifier (EDFA) booster type so that the maximum output power is 0.5 Watts. Another advantage of CW signal amplification is a signal with lower fluctuations compared to a situation where the pulse signal is amplified. The amplified CW signal is then modulated in the acousto-optic modulator (AOM) to 200 ns pulses, with a repetition rate given by the total length of fiber under test. If we consider the total length of approximately 40 km, then the repetition rate is given as follows:

(1) $$\tau =\frac{2\times L}{c/n}=\frac{2\times 40\times 10^{3}}{3\times 10^{8}/1.46}=3.89\times 10^{-4}\;\;\;\;\;\;(s),$$

and the repetition rate is then as follows:

(2) $$f=\frac{1}{\tau }=\frac{1}{3.89\times 10^{-4}}=2.57\;\;\;\;\;\;(\mathrm{kHz}).$$

Based on definition of peak energy,

(3) $$P_{peak}=\frac{E}{\Delta t},$$

we can calculate the total pulse energy, from which average power is given by the following equation:

(4) $$P_{avg}=\frac{E}{T}=Ef\approx 2.5\times 10^{-4}\;\;\;\;\;\;\left[W\right].$$

**Figure 1:**
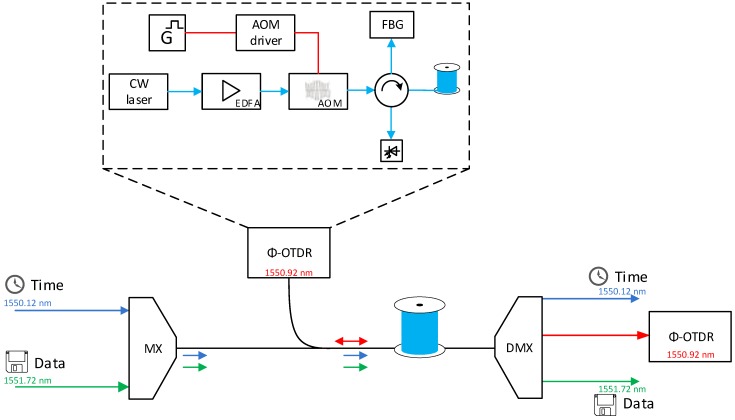
Measurement setup.

Before the pulse signal is coupled through the 3rd port of the circulator to the optical fiber, the amplified spontaneous emission (ASE) is filtered out using the fiber Bragg grating (FBG) mirror on port 2 of the circulator. The backscattered signal is detected on the 4th port of the circulator, and before the opto-electric conversion in the photodetector, the signal is amplified in the low-sensitivity EDFA preamplifier. Our measurement setup is shown in Figure 1.

### 3.2. Accurate Time Measurement Method

**Figure 2:**
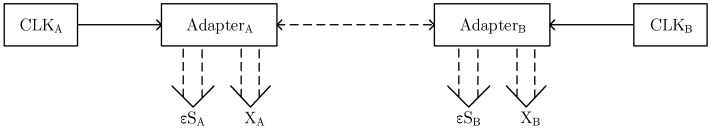
Home-developed time adapter.

Systems developed for highly accurate time scale comparison utilize a method based on a bidirectional transmission with a symmetrical transport delay in both directions [54,55]. The principle can be seen in Figure 2.

**Figure 3:**
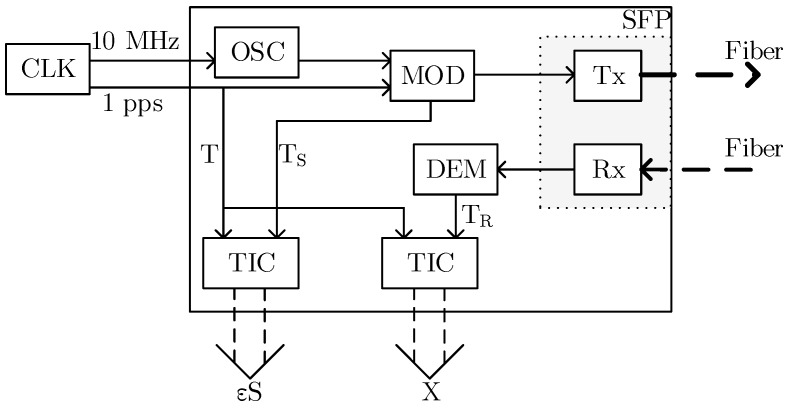
Home-developed time adapter.

The two adapters are connected by a two-way optical line. Each of the adapters is provided with the 1PPS timing signal and 10 MHz frequency from a local clock at its input, and two electrical signals are internally generated, specifically $T_{Ri}$ (*i* = A, B), representing the received and decoded 1PPS signal from the remote adapter, and $T_{Si}$, representing the moment when the encoded 1PPS signal is sent to the optical line. The $T_{Si}$ and $T_{Ri}$ signals are fed into two time interval counters (TICs) that are started by the $T_{i}$ signal—the 1PPS input signal. The first TIC is used to measure the interval $x_{i}$ between $T_{i}$ and $T_{Ri}$ (the difference between a local and a remote 1PPS). The second TIC is used to measure the delay in the $\varepsilon_{Si}$ adapter, i.e., the time between the $T_{i}$ and $T_{Si}$ signals. The internal structure of the adapter is shown in Figure 3.

The 1PPS pulse from the local clock arrives at adapter A at time $t_{A}$. It is then sent to the optical line at time $t_{SA}$ and received by adapter B at time $t_{RB}$. By analogy, 1PPS from the remote clock arriving at adapter B at time $t_{B}$ is sent at $t_{SB}$ and received by adapter A at time $t_{RA}$. Thus, $\Theta_{AB}=t_{B}-t_{A}$ is the offset of both clocks, $\varepsilon_{Si}=t_{Si}-t_{i};i=A,B$ is the delay within the adapter *i*, and $\delta_{AB}=t_{RB}-t_{SA}$ (can also be defined as $\delta_{BA}=t_{RA}-t_{SB}$) is the line delay from A to B (B to A).

By using TICs outputs (two in each location), it is possible to measure the internal delay of the $\varepsilon_{Si}$ adapter and the following time intervals:

(5) $$x_{A}=t_{RA}-t_{A}=\Theta_{AB}+\varepsilon_{SB}+\delta_{BA},$$

(6) $$x_{B}=t_{RB}-t_{B}=-\Theta_{AB}+\varepsilon_{SA}+\delta_{AB}.$$

In the case of a symmetric line, the delay δ in both directions will be the same, specifically: $\delta =\delta_{AB}=\delta_{BA}$. In a real optical path, we can either transmit the signal unidirectionally with the same wavelength in a pair of optical fibers or bidirectionally in a single optical fiber at different wavelengths. In the first case, there will be a challenge with an asymmetry of the physical length of the fibers; in the latter case, the optical propagation velocity will slightly differ, as it is given by the coefficient of chromatic dispersion, which generally depends on the wavelength. In both of these cases, there will be an asymmetry of delay Δ:

(7) $$\Delta =\delta_{BA}-\delta_{AB}$$

which needs to be computed in the case of a bidirectional transmission in a single fiber or evaluated during the calibration phase of the transmission route. Therefore, the time offset of the clock is given as follows:

(8) $$\Theta_{AB}=\frac{\left(\left(x_{A}-x_{B}\right)+\left(\varepsilon_{SA}-\varepsilon_{SB}\right)-\Delta \right)}{2}$$

By using TICs outputs (two in each location), it is possible to measure the internal delay of the $\varepsilon_{Si}$ adapter and the following time intervals:

(9) $$x_{A}=t_{RA}-t_{A}=\Theta_{AB}+\varepsilon_{SB}+\delta_{BA},$$

(10) $$x_{B}=t_{RB}-t_{B}=-\Theta_{AB}+\varepsilon_{SA}+\delta_{AB}.$$

In the case of a symmetric line, the delay δ in both directions will be the same, specifically: $\delta =\delta_{AB}=\delta_{BA}$. In a real optical path, we can either transmit the signal unidirectionally with the same wavelength in a pair of optical fibers or bidirectionally in a single optical fiber at different wavelengths. In the first case, there will be a challenge with the asymmetry of the physical length of the fibers; in the latter case, the optical propagation velocity will slightly differ, as it is given by the coefficient of chromatic dispersion, which generally depends on the wavelength. In both of these cases, there will be an asymmetry of delay Δ:

(11) $$\Delta =\delta_{BA}-\delta_{AB}$$

which can be calculated in the case of a bidirectional transmission in a single fiber. The difference in propagation times is shown in Equation (12). *L* is the fiber length, *d* is chromatic dispersion coefficient and D is the chromatic dispersion of passive components.

(12) $$\Delta =\left(dL+D\right)\Delta \lambda \;$$

As mentioned above, the wavelength and polarization sensitivity of the refractive index need to be taken into account. For example, *L* = 95 km, *d* = 17.5 ps/nm/km, Δλ = 0.8 nm, and *D* = 200 ps/nm results in the propagation time difference of 1.49 ns. Otherwise, in the case of the time transfer in a pair of unidirectional fibers, there is no possibility of calculating the delay asymmetry, as we cannot guarantee the same physical length of both fibers—the delay asymmetry must be evaluated during the phase of the calibration of the transmission route. Therefore, the time offset of the clock is given as follows:

(13) $$\Theta_{AB}=\frac{\left(\left(x_{A}-x_{B}\right)+\left(\varepsilon_{SA}-\varepsilon_{SB}\right)-\Delta \right)}{2}$$

### 3.3. Coriant Groove

Coriant Groove${}^{TM}$ G30 represents the network disaggregation platform. The increasing traffic of streaming, cloud content delivery, and data transmission has provided an opportunity for vendors. The Coriant Groove G30 is an open-line system (OLS) with industry-leading density, flexibility, and low power consumption. It supports a line capacity up to 4.8 Tbps in a 1U rack mount. We use Coriant as a data source with 100 Gbps transmission speed. On the other hand, Coriant provides 100 G, 150 G, and 200 G in different modulation formats, such as dual-polarization quadrature phase shift keying (DS-QPSK), dual polarization 8 quadrature amplitude modulation (DP-8QAM), and dual polarization 16 quadrature amplitude modulation (DP-16QAM), respectively.

## 4. Results and Discussion

During the measurement, different fibers were emphasized. Initial testing was conducted using a photonic service (accurate time), sensory system, and data transfer. Each of the services was defined by its own wavelength. Measurement of the bit error rate was performed automatically using Coriant Groove™ G30 in a 1 min period. The authors aimed at demonstrating the possibilities of data flow being influenced by the pulse sensory system. It can be assumed that low power duration will result in the availability of a service throughout the testing, whereas a gradual increase in pulse duration will eventually affect the data signal. The data signal was not the only service being monitored. The accurate time transmission allows for evaluating the delay per fiber. Optical fibers are defined by their type and refractive index. After each of the optical fibers was measured, a combination of G.652D and G.653 fibers was created. The measured values of pre-forward error correction-bit error rate (pre-FEC-BER) indicate the error rate prior to applying any correction algorithm.

**Figure 4:**
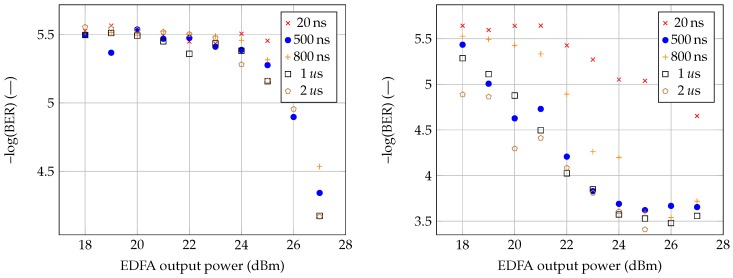
Measurement results for G.652D (on the **left** side) and G.653 fiber (on the **right** side).

Figure 4 shows the results for G.652D and G.653 fibers. As seen, the associated pre-FEC-BER values are independent and show a certain scattering pattern. However, linear dependence cannot be assumed at this point. The scattering could theoretically be eliminated by using a longer measuring section. For our measurement, the section of one minute was chosen. Coriant Groove™ G30 supports measuring ranges of 1, 15, or 60 min. As more emphasis is put on proving an influence among the services, the use of longer sections was not necessary. The pre-FEC-BER values are indicative of the measurements. However, as seen in Figure 4, the values of pre-FEC-BER gradually decrease as the increasing power of the sensor system affects the data transmission. It should be pointed out that for the G.652D fiber, i.e., the standard telecommunication fiber, the accurate time transmission was stable for all tested pulse durations and output power values of the erbium-doped fiber amplifier (EDFA). The average time delay was ≈35,328.415 ns. On the right side of Figure 4, measurements of a single G.653 fiber are visualized. With the increasing sensor system’s power, the values of the pre-FEC-BER deteriorated by an order of magnitude in comparison to those of G.652D. The accurate time transmission dropped out immediately after increasing the sensing system’s power from 25 dBm to 26 dBm even for the shortest pulse, with a pulse duration of 20 ns. After extending the pulse duration from 20 to 500 ns, the accurate time transmission showed errors from the sensory system’s power of 23 dBm. Another increase in pulse duration up to 800 ns did not affect the accurate time transmission, as the transmission failure occurred again after the sensory system’s power increased from 22 to 23 dBm. Next, the pulse duration was raised again to 1 μs, where the accurate time transmission showed frequent errors (no delay value displayed) from the power of 22 dBm. This trend is reflected by decreasing the pre-BER-FEC values on Coriant Groove™ G30, as seen in Figure 4 (on the right side). As a result, G.653 fiber was found to be the least appropriate to conduct simultaneous transmission of photonic services. The average accurate transmission time delay was ≈34,968,292 ns.

**Figure 5:**
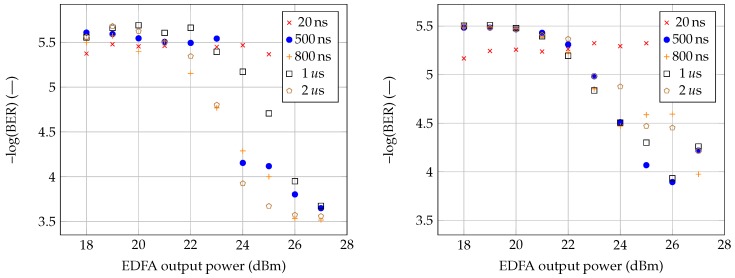
Measurement results for G.655 (on the **left** side) and a combination of G.652D and G.6533 fibers (on the **right** side).

The previous two fibers had the same length of 7 km. In comparison, G.655 fiber has a length of 10 km. The results for the G.655 fiber measurement are very similar to those of G.652D. Figure 5 (on the left side) shows the measured pre-FEC-BER values for the G.655 fiber. In comparison with the previous examples, the largest scattering is found by using this particular fiber. As mentioned above, these errors are caused by the measurement methodology when a very short section was chosen to measure the data sequence during the evaluation. None of the tested pulse durations disturbed the accurate time transfer during the measurement, and there were no errors such as those found for the G.653 fiber. With the maximum power of the sensor system and pulse durations of 500, 800, 1000, and 2000 ns, comparable pre-FEC-BER values were achieved. The last connection was made by combining G.652D and G.653 fibers. From the results (see Figure 4), it is clear that the sensor system could not affect any of the services in the G.652D fiber. In comparison with the G.653 fiber, the disturbance of the accurate time transmission appeared even when the shortest pulse of the sensor system was used. For this reason, a combination of both fibers was created since the current optical paths can be made up of individual sections, and the use of different types of fiber is not excluded. Keeping the parameters stable across the optical path is essential for the operation of photonic services. With the shortest pulse being used, there were no failures of the accurate time transmission. However, by increasing the pulse duration to 500 ns, occasional errors did occur. With a further increase in the power to 28 dBm, such errors increasingly occurred more frequently. The average accurate time delay was ≈70,234,602 ns.

The time and data signals were transmitted through the fiber without further amplification, i.e., at normal power levels (max +6 dBm output power from transceiver). Since the sensory system’s power output was more than 20 dBm, the performance of the data and time transmission were affected. Nonlinear phenomena caused by the high-power pulse signal induced the instability, and the data signal waveform fluctuates over time, which results in data errors. Increasing the channel spacing should lead to BER improvement because of the lower interaction of transmitted signals. The minor difference in BER (as seen in Figure 4 and Figure 5) for different pulse durations is related to measurement accuracy. Moreover, greater accuracy would be achieved by a longer measurement time, but even a selected measurement time of 1 min yields sufficient results to determine whether the 100 GHz channel spacing defined by ITU 694.1 is sufficient or not.

## 5. Conclusions

This article introduces the concept of photonic service and its definition. To use photonic services, it is crucial to keep their parameters stable with regard to the reserved bandwidth across the spectrum throughout the optical path. The transmission of an accurate time and a stable frequency is often described and implemented separately, although these are special applications that have very specific requirements to be fulfilled.

Next, we also presented our own sensor system based on the principle of phase OTDR, as well as a system for accurate time transmission. Both of these systems were used during the measurements. As the WDM principle has been known for several decades, we can expect an increase in the use of measuring the simultaneous transmission of photonic services in a single fiber. However, photonic service considerations are no longer only about the transmission of data signals or special applications. In the future, it will be necessary to take into account the deployment of sensor systems for critical infrastructure protection. Infrastructure protection is currently a highly sought after topic in the Czech Republic and worldwide, and with the falling prices of optical fiber sensors, this trend is likely to continue.

Although the ITU grid defines the 100 GHz spacing between services as sufficient, our measurements show the exact opposite. The spacing between services without a sensor system is quite sufficient; however, with the use of high-power sensor systems, service interaction may occur. Our results also show that the influence among the services is also demonstrated by the type of optical fiber being used. While G.652D and G.655 fibers are considerably less susceptible to service interactions, if critical infrastructure is made up of G.653 fibers, higher service spacing should definitely be considered.

Our future work will continue with another higher modulation format with data transmission (150G with DP-8QAM and 200G with DP-8QAM), and we will also address a stable frequency evaluation and a higher output power of a sensor system.

## Abbreviations

The following abbreviations are used in this manuscript:

AOM	Acousto-optic modulator
ASE	Amplified spontaneous emission
BER	Bit error rate
CW	Continuous wave
CWDM	Coarse wavelength division multiplex
DP-8QAM	Dual-polarization 8 quadrature amplitude modulation
DP-QPSK	Dual-polarization quadrature phase shift keying
DWDM	Dense wavelength division multiplex
EDFA	Erbium-doped fiber amplifier
FBG	Fiber Bragg grating
GMT	Greenwich mean time
GNSS	Global Navigation System Satellite
HD	High definition
IEEE	Institute of Electrical and Electronics Engineers
IPTV	Internet protocol television
UHD	Ultrahigh definition
ITU	International Telecommunication Union
NREN	National Research and Education Network
NTP	Network time protocol
OLS	Open-line system
Φ-OTDR	Phase-sensitive optical time domain reflectometer
pre-FEC-BER	Pre-forward error correction-bit error rate
PTP	Precision time protocol
RF	Radio frequency
SDM	Space division multiplex
SI	International System of Units
TAI	International atomic time
TIC	Time interval counter
TWSTFT	Two-way satellite time and frequency transfer
USA	United States of America
UDP	User datagram protocol
UTC	Coordinated universal time
VoD	Video on demand
VLBI	Very-long-baseline interferometry
WDM	Wavelength division multiplexing
WR	White Rabbit

## References

[1] Cisco Visual Networking Index: Forecast and Trends, 2017–2022. https://bit.ly/2TYstY8.

[2] Mikaeil A.M., Hu W., Ye T., Hussain S.B. (2017). Performance Evaluation of XG-PON Based Mobile Front-Haul Transport in Cloud-RAN Architecture. J. Opt. Commun. Netw..

[3] Horvath T., Munster P., Oujezsky V., Vojtech J. (2018). Activation Process of ONU in EPON/GPON/XG-PON/ NG-PON2 Networks. Appl. Sci..

[4] Fu S., Zeng L., Ji R., Grillanda S., Morichetti F., Carminati M., Sampietro M., Dentin A., Dede A., Vannucci A. (2016). Automatic control of the silicon microring OSR and multiplexer in DML-based WDM transmitter for 40G TWDM-PON OLT. Proceedings of the 2016 IEEE 13th International Conference on Group IV Photonics (GFP).

[5] Horvath T., Munster P., Cymorek P., Oujezsky V., Vojtech J. (2017). Implementation of NG-PON2 transmission convergence layer into OPNET modeler. Proceedings of the 2017 International Workshop on Fiber Optics in Access Network (FOAN).

[6] Tian M., Wang L., Chen X., Liu Y. (2013). The investigation on 40G long reach coherent passive optical network. Proceedings of the 2013 3rd International Conference on Consumer Electronics, Communications and Networks.

[7] Suzuki N., Miura H., Matsuda K., Matsumoto R., Motoshima K. (2017). 100G to 1T based Coherent PON Technology. Proceedings of the 2017 European Conference on Optical Communication (ECOC).

[8] Sugimoto S., Minemura K., Kobayashi K., Seki M., Shikada M., Ueki A., Yanase T., Miki T. (1977). High-speed digital-signal transmission experiments by optical wavelength-division multiplexing. Electron. Lett..

[9] Tomlinson W.J., Lin C. (1978). Optical wavelength-division multiplexer for the 1–1.4 μm spectral region. Electron. Lett..

[10] Conradi J., Maciejko R., Straus J., Few I., Duck G., Sinclair W., Springthorpe A.J., Dyment J.C. (1981). Laser based WDM multichannel video transmission system. Electron. Lett..

[11] Wu K.-L. (1999). An optimal circular-waveguide dual-mode filter without tuning screws. IEEE Trans. Microw. Theory Tech..

[12] Judy A.F. (1997). Optimizing fiber dispersion for DWDM systems. Proceedings of the Optical Fiber Communication Conference.

[13] G.652: Characteristics of a Single-Mode Optical Fibre and Cable. https://www.itu.int/rec/T-REC-G.652-201611-I/en.

[14] Chen X., Zeng J., Zhao J., Wang Z., Liu H. (2016). A precise time-integration for analysis of time-domain response to lossy transmission lines. Proceedings of the 2016 9th International Congress on Image and Signal Processing, BioMedical Engineering and Informatics (CISP-BMEI).

[15] Vojtech J., Slapak M., Skoda P., Radil J., Havlis O., Altmann M., Munster P., Velc R., Kundrat J., Altmannova L. (2017). Joint accurate time and stable frequency distribution infrastructure sharing fiber footprint with research network. Opt. Eng..

[16] Ye Y., Spina D., Xing Y., Bogaerts W., Dhaene T. (2018). Fast and Accurate Time-Domain Simulation of Passive Photonic Systems. Proceedings of the 2018 International Conference on Electromagnetics in Advanced Applications (ICEAA).

[17] Narbonneau F., Lours M., Lopez O., Daussy C., Chambon S.D., Bize S., Klein A.A., Chardonnet C., Clairon A., Santarelli G. (2004). Ultra-stable ground frequency dissemination via optical fibres. Proceedings of the 18th European Frequency and Time Forum (EFTF 2004).

[18] Xu D., Lee W.K., Stefani F., Pottie P.-E., Amy-Klein A., Lopez O. (2017). Hybrid optical link for ultra-stable frequency comparison. Proceedings of the 2017 Joint Conference of the European Frequency and Time Forum and IEEE International Frequency Control Symposium (EFTF/IFC).

[19] Wlodarczyk P., Krehlik P., Sliwczyeski L. (2018). Comparison of highly-stable optical frequency transfer in a single bidirectional and double unidirectional fibers. Proceedings of the 2018 European Frequency and Time Forum (EFTF).

[20] Bao N.-H., Wu Y.-K., Su G.-Q., Yuan Y., Luo D.-Y. (2017). Hierarchical fairness based re-provisioning in post-disaster telecom networks. Proceedings of the 2017 International Conference on Computer, Information and Telecommunication Systems (CITS).

[21] Cheng Z., Zhang X., Shen S., Yu S., Ren J., Lin R. (2018). T-Trail: Link Failure Monitoring in Software-Defined Optical Networks. J. Opt. Commun. Netw..

[22] Bao N.-H., Luo D.-Y., Chen J.-B. (2018). Reliability threshold based service bandwidth recovery scheme for post-disaster telecom networks. Opt. Fiber Technol. (OFT).

[23] Udd E. (1995). An overview of fiber-optic sensors. Rev. Sci. Instrum..

[24] Hartog A.H. (2017). An Introduction to Distributed Optical Fibre Sensors (Series in Fiber Optic Sensors).

[25] Chung Y., Jin W., Lee B., Canning J., Nakamura K., Yuan L., Wang Z., Jia X., Wu H., Peng F. (2017). Towards ultra-long-distance distributed fiber optic sensing. Proceedings of the 2017 25th Optical Fiber Sensors Conference (OFS).

[26] Munster P., Vojtech J., Horvath T., Havlis O., Hanak P., Cucka M., Filka M. (2016). Simultaneous transmission of distributed sensors and data signals. Proceedings of the 2016 39th International Conference on Telecommunications and Signal Processing (TSP).

[27] Munster P., Horvath T., Vojtech J., Havlis O., Slapak M., Skoda P., Radil J., Velc R., Hula M. (2017). Interference of Data Transmission in Access and Backbone Networks by High-Power Sensor System. Fiber Integr. Opt..

[28] Ludlow A.D., Boyd M.M., Ye J., Peik E., Schmidt P.O. Optical Atomic Clocks. https://arxiv.org/pdf/1407.3493.pdf.

[29] Bloom B.J., Nicholson T.L., Williams J.R., Campbell S.L., Bishof M., Zhang X., Zhang W., Bromley S.L., Ye J. (2014). An optical lattice clock with accuracy and stability at the 10^18^ level. Nature.

[30] Bordé C.J. (2005). Base units of the SI, fundamental constants and modern quantum physics. Philos. Trans. R. Soc. A Math. Phys. Eng. Sci..

[31] Rosenband T., Hume D.B., Schmidt P.O., Chou C.W., Brusch A., Lorini L., Oskay W.H., Drullinger R.E., Fortier T.M., Stalnaker J.E. (2008). Frequency Ratio of Al+ and Hg+ Single-Ion Optical Clocks; Metrology at the 17th Decimal Place. Science.

[32] Chou C.W., Hume D.B., Rosenband T., Wineland D.J. (2010). Optical Clocks and Relativity. Science.

[33] Jiang Z., Lin S.-Y., Tseng W.-H. (2017). Fully and optimally use the redundancy in a TWSTFT network for accurate time transfer. Proceedings of the 2017 Joint Conference of the European Frequency and Time Forum and IEEE International Frequency Control Symposium (EFTF/IFC).

[34] Weiss M. (2017). Getting accurate time from GNSS receivers: Considerations to approach nanosecond time. Proceedings of the 2017 IEEE International Symposium on Precision Clock Synchronization for Measurement, Control, and Communication (ISPCS).

[35] Prochazka I., Yang F. (2009). Photon counting module for laser time transfer via Earth orbiting satellite. J. Mod. Opt..

[36] Giorgetta F.R., Swann W.C., Sinclair L.C., Baumann E., Coddington I., Newbury N.R. (2013). Optical two-way time and frequency transfer over free space. Nat. Photonics.

[37] Ebenhag S.-C., Hedekvist P.O., Jarlemark P., Emardson R., Jaldehag K., Rieck C., Lothberg P. (2010). Measurements and Error Sources in Time Transfer Using Asynchronous Fiber Network. IEEE Trans. Instrum. Meas..

[38] Rost M., Piester D., Yang W., Feldmann T., Wübbena T., Bauch A. (2012). Time transfer through optical fibres over a distance of 73 km with an uncertainty below 100 ps. Metrologia.

[39] Newbury N.R. (2015). Frequency and Timing Distribution using Optical Methods. CLEO: 2015.

[40] Vojtech J., Smotlacha V., Radil J. (2014). All optical two-way time transfer in strongly heterogeneous networks. Proceedings Volume 9202, Photonics Applications for Aviation, Aerospace, Commercial, and Harsh Environments V.

[41] Vojtech J., Smotlacha V., Skoda P., Kuna A., Hula M., Sima S., Ardanuy P.E., Puschell J.J., Bloom H.J. (2012). Photonic services, their enablers and applications. Proceedings of the SPIE Optical Engineering + Applications.

[42] Sliwczynski Ł., Krehlik P., Buczek Ł., Lipinski M. (2012). Frequency Transfer in Electronically Stabilized Fiber Optic Link Exploiting Bidirectional Optical Amplifiers. IEEE Trans. Instrum. Meas..

[43] Lopez O., Kanj A., Pottie P.-E., Rovera D., Achkar J., Chardonnet C., Amy-Klein A., Santarelli G. (2013). Simultaneous remote transfer of accurate timing and optical frequency over a public fiber network. Appl. Phys. B.

[44] Calonico D., Bertacco E.K., Calosso C., Clivati C., Godone A., Frittelli M., Mura A., Zucco M., Levi F., Costanzo G.A. (2014). Optical frequency transfer with a 1284 km coherent fiber link. Proceedings of the 2014 European Frequency and Time Forum (EFTF).

[45] Zhang H., Wu G., Hu L., Li X., Chen J. (2015). High-Precision Time Transfer over 2000-km Fiber Link. IEEE Photon. J..

[46] Vojtech J., Smotlacha V., Skoda P. (2015). Simultaneous transmission of accurate time in parallel with stable optical frequency in real fibre network over 612 km. Proceedings of the 2015 Optoelectronics Global Conference (OGC).

[47] Lopez O., Haboucha A., Chanteau B., Chardonnet C., Amy-Klein A., Santarelli G. (2012). Ultra-stable long distance optical frequency distribution using the Internet fiber network. Opt. Express.

[48] Wang B., Gao C., Chen W.L., Miao J., Zhu X., Bai Y., Zhang J.W., Feng Y.Y., Li T.C., Wang L.J. (2012). Precise and Continuous Time and Frequency Synchronisation at the 5· 10^−19^ Accuracy Level. Sci. Rep..

[49] Lopez O., Chardonnet C., Amy-Klein A., Kanj A., Pottie P.-E., Rovera D., Achkar J., Santarelli G. (2013). Simultaneous remote transfer of accurate timing and optical frequency over a public fiber network. Proceedings of the 2013 Joint European Frequency and Time Forum & International Frequency Control Symposium (EFTF/IFC).

[50] Lopez O., Chanteau B., Bercy A., Nicolodi D., Zhang W., Argence B., Abgrall M., Haboucha A., Kanj A., Rovera D. (2013). Ultra-stable long distance optical frequency distribution using the Internet fiber network and application to high-precision molecular spectroscopy. J. Phys. Conf. Ser..

[51] Munster P., Radil J., Vojtech J., Havlis O., Horvath T., Smotlacha V., Skaljo E. (2017). Simultaneous transmission of the high-power phase sensitive OTDR, 100Gbps dual polarisation QPSK, accurate time/frequency, and their mutual interferences. Proceedings Volume 10208, Fiber Optic Sensors and Applications XIV.

[52] Horvath T., Munster P., Vojtech J., Velc R., Oujezsky V. (2018). Simultaneous transmission of accurate time, stable frequency, data, and sensor system over one fiber with ITU 100-GHz grid. Opt. Fiber Technol..

[53] Munster P., Horvath T., Havlis O., Vojtech J., Radil J., Velc R., Skaljo E. (2017). Simultaneous transmission of standard data, precise time, stable frequency and sensing signals and their possible interaction. Proceedings Volume 10231, Optical Sensors 2017.

[54] Dostal J., Smotlacha V. (2015). Next generation of architecture for precise time measurements. Proceedings of the 2015 IEEE East-West Design & Test Symposium (EWDTS).

[55] Smotlacha V., Vojtech J. (2015). Accurate time distribution using optical fiber. Proceedings of the 2015 International Association of Institutes of Navigation World Congress (IAIN).

